# Portal Hypertension and a Stiff Liver

**DOI:** 10.7759/cureus.2768

**Published:** 2018-06-08

**Authors:** Felicia D'Alitto, Amina Scherz, Cristina Margini, Hendrik Von Tengg-Kobligk, Matteo Montani, Thomas Pabst, Annalisa Berzigotti

**Affiliations:** 1 Swiss Liver Center, Hepatology, University Clinic for Visceral Surgery and Medicine, Inselspital, University of Bern, Switzerland; 2 Department of Medical Oncology, Inselspital, University of Bern, Switzerland; 3 Department of Diagnostic, Interventional and Pediatric Radiology, Inselspital, University of Bern, Switzerland; 4 Institute of Pathology, University of Berne, Switzerland; 5 Department of Medical Oncology, Inselspital, University of Bern, Switzerland, USA; 6 Hepatology, University Clinic for Visceral Surgery and Medicine, Inselspital, University of Bern, Bern, CHE

**Keywords:** liver cirrhosis, amyloidosis, multiple myeloma, liver biopsy, elastography, hvpg

## Abstract

Portal hypertension (PH) is a common clinical syndrome leading to severe complications. In the western world, about 90% of cases of PH are due to liver cirrhosis, and thanks to the availability of ultrasound elastography methods, this diagnosis is usually confirmed at bedside. We report a case of a patient presenting with PH and ascites initially suspected of suffering from liver cirrhosis. The finding of large hepatomegaly and a massive increase in liver stiffness prompted us to perform a liver biopsy. This revealed no fibrosis, but diffuse primary amyloidosis (AL amyloidosis). We discuss the diagnostic and treatment of this case, with emphasis on non-invasive imaging methods available for diagnosis and follow up.

## Introduction

Portal hypertension (PH) is a common syndrome featuring severe clinical complications such as ascites and gastrointestinal bleeding due to gastroesophageal varices rupture. The most frequent cause of PH in the Western world is liver cirrhosis, which accounts for over 90% of cases. In patients presenting with signs of PH, ultrasound and ultrasound elastography are usually the first diagnostic tests performed, given their high sensitivity and specificity for the diagnosis of cirrhosis [1-2]. In particular, a high liver stiffness is considered an accurate sign of cirrhosis in the context of PH [1-2]. However, a non-expert interpretation of non-invasive diagnostic tests can lead to incorrect diagnosis, and the integration of invasive and non-invasive methods is key for an accurate assessment of this clinical syndrome in order to diagnose rarer causes. Here, we report a clinical case of a patient presenting with PH and increased liver stiffness, initially considered associated with liver cirrhosis but subsequently diagnosed with primary systemic amyloidosis in the context of multiple myeloma. We describe and discuss the use of invasive and non-invasive tests to achieve the final diagnosis and to follow-up the patient.

## Case presentation

A 60-year-old man was referred to our center in October 2015 due to ascites and increased liver enzymes ongoing since six months (Table 1).

**Table 1:** Laboratory tests on admission and after 18 months VGPR: very good partial response.

	Baseline	After 18 months (ongoing VGPR)	Reference normal values
Hemoglobin (g/L)	125	121	135 - 168
Hematocrit	0.38	0.37	0.40 - 0.50
MCV (fL)	105	91	80 - 98
Leucocytes (G/L)	6.6	4.96	3.5 - 10.5
Platelets (G/L)	222	148	140 - 380
Plasma Creatinine (µmol/L)	93	101	59 - 104
eGFR according to CKD-EPI (mL/min)	77	68	> 59
Sodium (mmol/L)	140	143	136 - 145
Potassium (mmol/L)	3.9	4.4	3.5 - 4.5
Plasma protein (g/L)	58	70	64 - 83
Albumin (g/L)	32	40	35 - 52
IgG (g/L)	7.12	7.41	7.00 - 16.00
Total Bilirubin (µmol/L)	20	5	< 17
ASAT (U/L)	75	34	< 50
ALAT (U/L)	35	33	< 50
Alk. Phosphatase (U/L)	181	65	40 - 130
Glutamyl transferase (U/L)	244	50	< 60
INR ratio	1.31	<1.0	
Quick (%)	57	>100	70 - 130

He had no previous history of liver disease and he complained of fatigue and weight loss (16 Kg) over eight months. The patient referred alcohol consumption of about 50 g/day in the last 30 years, which he stopped on the month before admission when ascites was noted. He had no history of chronic diseases except a known allergy to eggs and soy proteins.

**Figure 1:**
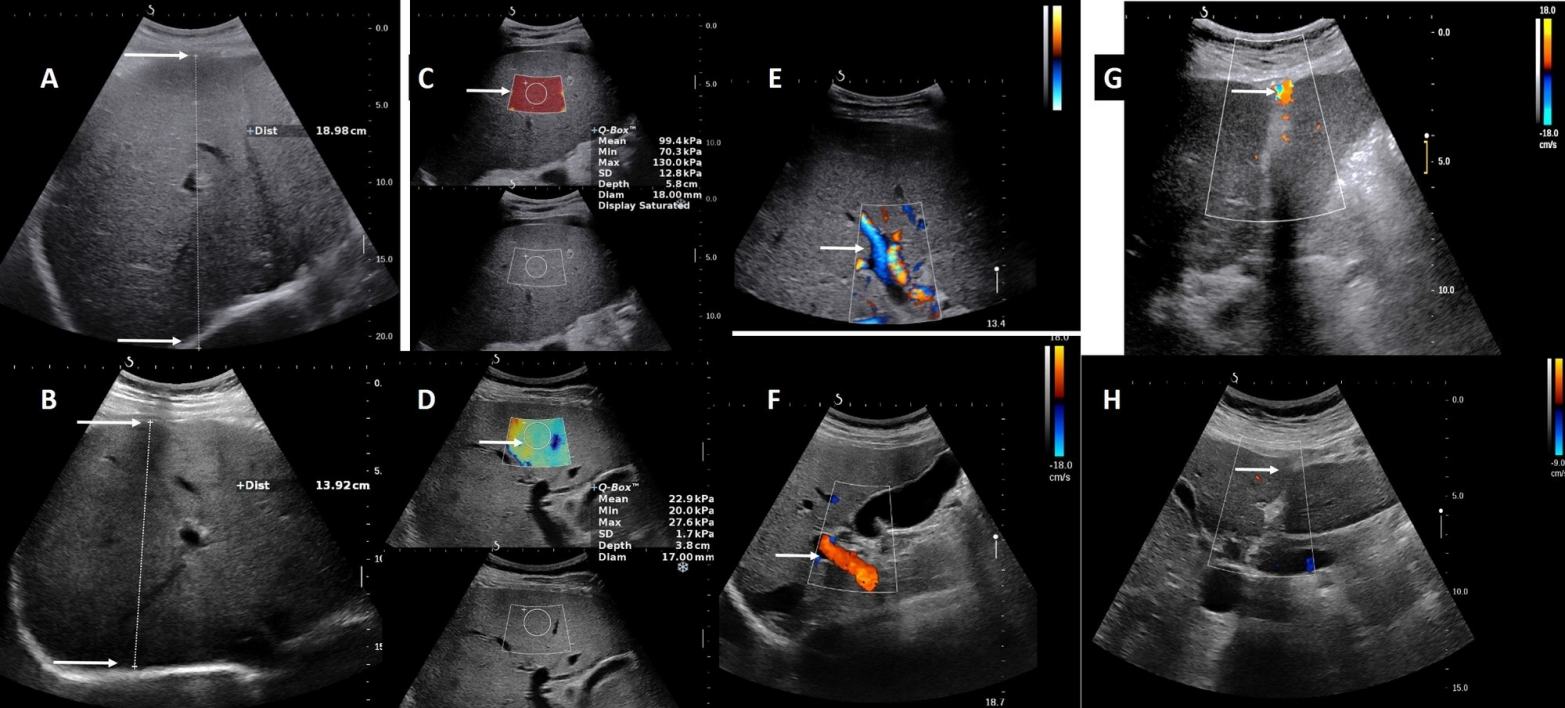
Ultrasound and 2D-SWE findings at presentation (October 2015, upper row) and 18 months after diagnosis (ongoing VGPR to chemotherapy; March 2017, lower row) The antero-posterior diameter of the right liver lobe measured at the mid-clavicular line was markedly enlarged on presentation (Panel A, arrow) and decreased on VGPR (Panel B, arrow). Similarly, liver stiffness by 2D-SWE was very high on presentation (Panel C, arrow) and decreased markedly on VGPR (Panel D, arrow). Intrahepatic portal blood flow was reversed (hepatofugal) on presentation (Panel E, arrow) and returned to normality (hepatopetal) on VGPR (Panel F, arrow). In addition patency of paraumbilical vein was noted on presentations (Panel G, arrow), and was no longer seen on VGPR (Panel H, arrow). 2D-SWE: 2-dimensional shear wave elastography; VGPR: very good partial response.

On the examination at our center, the patient featured sarcopenia and ascites. Liver ultrasound showed a large hepato-splenomegaly with an irregular surface, a hyperechoic liver parenchyma, and signs of intrahepatic portal hypertension (patent paraumbilical vein; reversed portal venous flow) as well as ascites (Figure 1). No focal liver lesions were observed.

Liver stiffness measurement (LSM) was performed using two different ultrasound elastography techniques: transient elastography (TE); M probe (FibroScan, Echosens, Paris, France), and 2-dimensional shear wave elastography (2D-SWE); SC6–1 probe (Aixplorer ultrasound system, Supersonic Imagine, Aix-en-Provence, France). Both showed very high values, clearly above normality (Table 2) [1].

**Table 2:** Comparison of liver and spleen stiffness and size during admission and after 18 months VGPR: very good partial response; TE: transient elastography; 2D-SWE: two dimensional shear wave elastograpy.

	Technique	Baseline	After 18 months (ongoing VGPR)
LIVER STIFFNESS	TE (kPa)	75 ± 0 Success Rate 100%	35.3 ± 7.6 Success Rate 100%
	2D-SWE (kPa)	99.4 ± 12.8	22.9 ± 1.7
LIVER SIZE	Grey scale ultrasound measurement of the antero-posterior diameter at the right mid-clavicular line (cm)	18.9	13.9
SPLEEN STIFFNESS	TE (kPa)	45 ± 3.5 Success Rate 100%	37.4 ± 5.8 Success Rate 100%
	2D-SWE (kPa)	N/A	31.3 ± 3
SPLEEN SIZE	Grey scale ultrasound measurement of the bipolar diameter (cm)	14.4	13.5

**Figure 2:**
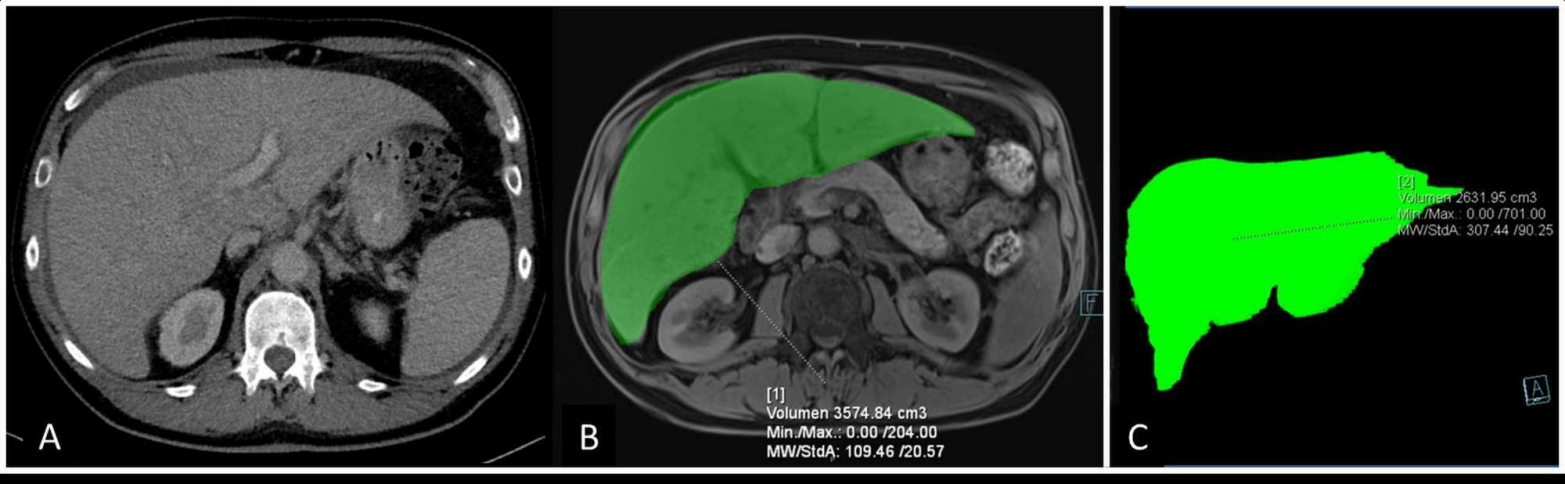
Findings on cross sectional imaging on presentation and during the follow-up Computed tomography (CT) scan on presentation showing a clearly enlarged liver (Panel A). Magnetic resonance imaging (MRI) in February 2016 showed a stable hepatomegaly (Panel B), which improved substantially on VGPR one year later (volumetric reconstruction of MRI liver images; Panel C). VGPR: very good partial response.

Spleen stiffness was measured by TE and demonstrated values compatible with portal hypertension (Table 2) [2]. A computed tomography (CT) scan confirmed the morphological imaging findings shown on ultrasound; CT-based volumetry of the liver and spleen reached high values of 3298 ml and 621 ml, respectively (Figure 2).

Esophagogastroduodenoscopy demonstrated small esophageal varices. A diagnostic paracentesis showed a high serum-ascites albumin gradient (2.1 g/dL), confirming a portal hypertension-related cause. Urinalysis showed a ++ proteinuria. All common causes of chronic liver disease (viral, autoimmune, metabolic) were excluded and a decision to perform liver biopsy was taken. Given the presence of portal hypertension, a transjugular liver biopsy was preferred in order to simultaneously measure the hepatic venous pressure gradient (HVPG) and to minimize the risk of intraperitoneal bleeding.

The procedure was performed by accessing the right jugular vein through Seldinger’s technique. The HVPG was 24 mmHg (wedged hepatic venous pressure 33 mmHg; free hepatic venous pressure nine mmHg), which confirmed a sinusoidal cause of portal hypertension. The liver biopsy was uneventful.

**Figure 3:**
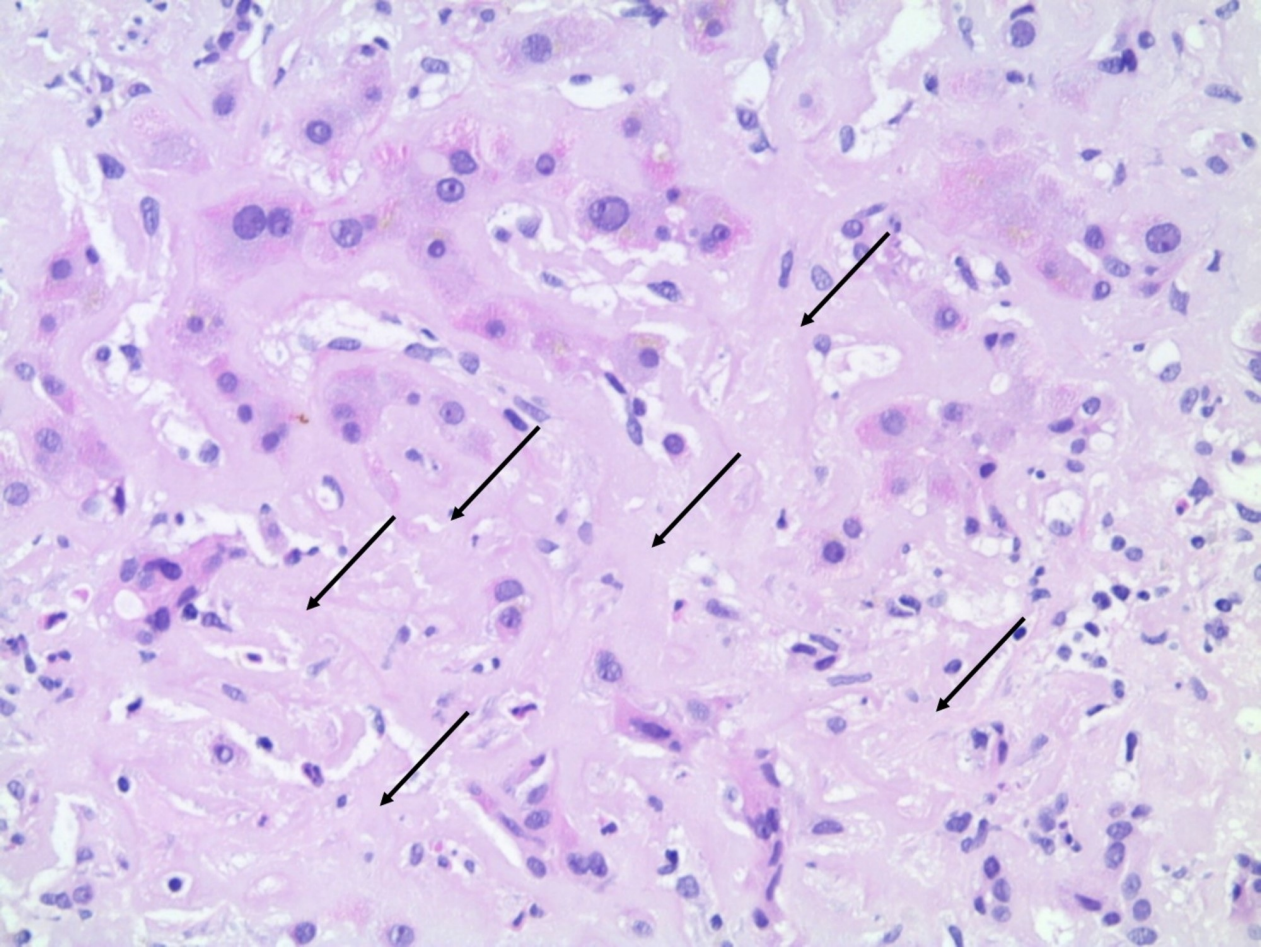
Liver histology on diagnosis Extensive amyloid deposition with obliteration of sinusoids was observed (arrows). Hematoxylin & Eosin 200x.

**Figure 4:**
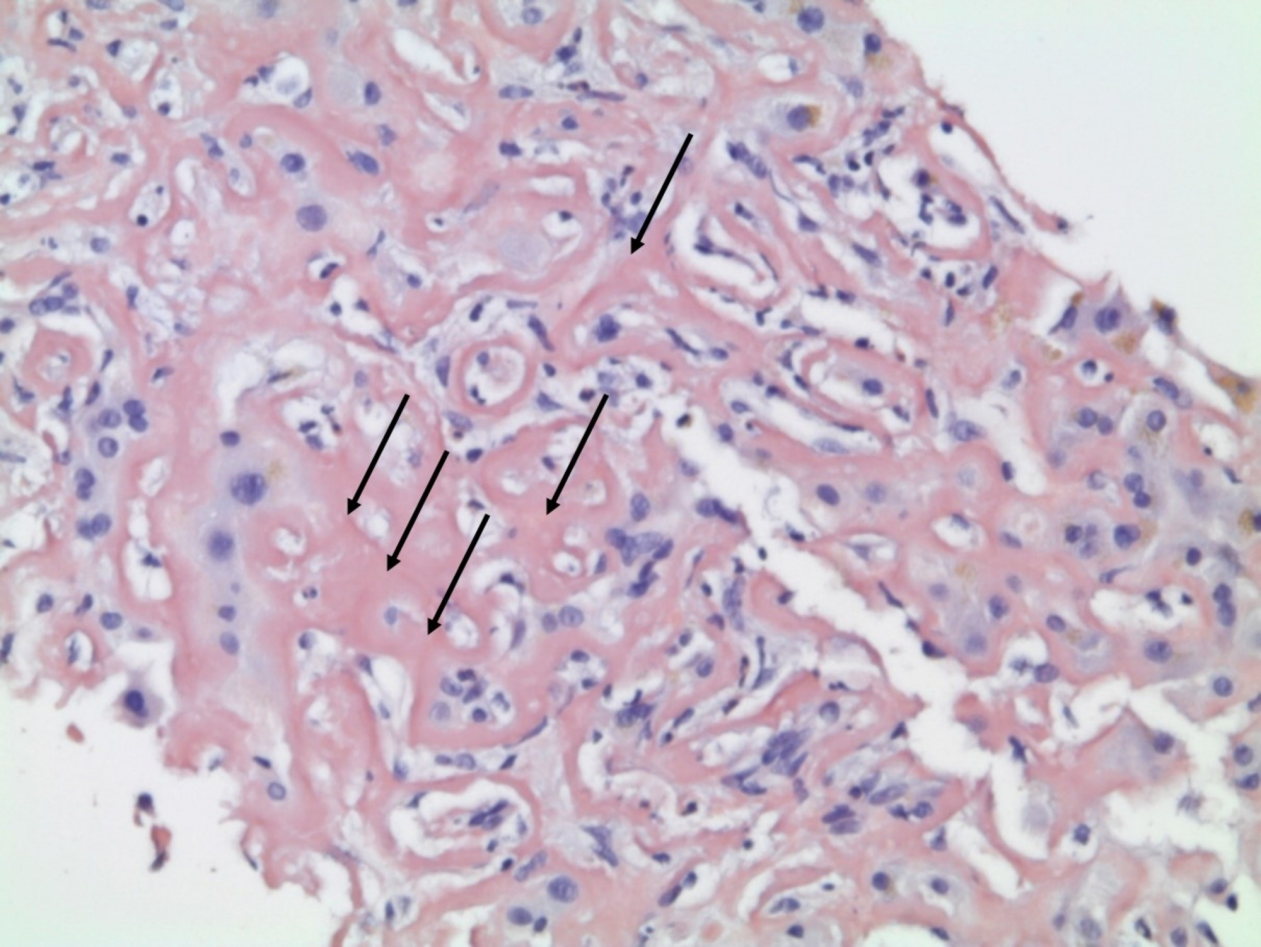
Liver histology on diagnosis Extensive amyloid deposition with obliteration of sinusoids was observed (arrows). Congo red 100x.

Liver histology showed massive amyloid deposition predominantly in the Dissé space with obliteration of sinusoids and trabecular atrophy, with only minimal portal and perisinusoidal fibrosis (Figures 3-4).

A bone marrow biopsy demonstrated mature cells plasmacellular myeloma with kappa immunophenotype and 20%-30% diffuse interstitial infiltration. Congo Red stain showed no amyloid infiltration.

Multiple myeloma with systemic primary amyloidosis (AL) with liver involvement was diagnosed and the patient was referred to the oncology department. Cardiac magnetic resonance imaging (MRI) and electrocardiogram (EKG) did not show features of cardiac involvement. Amyloidosis-related neuropathy affected the autonomous nervous system (relevant orthostatic hypotension) and the peripheral sensory nervous system (hypo- and dysesthesia in the distal upper and lower limbs). Due to the severe malnutrition and sarcopenia associated with amyloidosis and portal hypertension, the patient was put on parenteral nutrition.

From November 26, 2015 until February 23, 2016, the patient was treated with four cycles of a first line regimen according to the CyBorD scheme, comprising cyclophosphamide 350 mg/m2 intravenous (iv) on days one, eight, and fifteen; 1.0 mg/m2 bortezomib subcutaneous (sc) on days one, four, eight, and eleven; and dexamethasone 20 mg per os (po) on days one, four, eight, and eleven; within a 21 days schedule.

The treatment was haematologically well tolerated, and orthostatic hypotension resolved completely in the fourth cycle, whereas the patient developed fatigue (CTCAE grade two), loss of appetite, and aggravation of peripheral sensory neuropathy (PNP CTCAE grade two, pain); the latter was assumed as bortezomib-triggered. During the course of chemotherapy, he also developed severe complications of portal hypertension including refractory ascites requiring multiple large-volume paracentesis, bacterial infections, and hepato-renal syndrome.

After the four therapy cycles, the remission status indicated a very good partial response (VGPR), with a difference between involved and uninvolved serum free light chains (DFLC) of 21 mg/L, compared to 258.6 mg/L at diagnosis. Abdominal MRI in February 2016 showed stable hepato- (3574 ml) and splenomegaly (613 ml) with minimal heterogeneity of the liver parenchyma in the T1w phase suggesting amyloid content.

Few weeks after completion of first line chemotherapy, fatigue and loss of appetite resolved completely; parenteral nutrition could be stopped. A few months later, the drug-induced PNP also improved to CTCAE grade one. The patient refused further consolidation of first-line treatment with high dose chemotherapy (HDCT) and autologous stem cell transplantation (ASCT) because of the side effects related to the induction therapy with CyBorD he previously experienced. No further episodes of clinical decompensation of liver disease were observed.

One year later (eighteen months after the diagnosis), the patient was in very good clinical condition, asymptomatic, without ascites and his liver tests improved (Table 1). On ultrasound, the liver was significantly smaller, with slightly heterogenous aspect of the parenchyma as seen in Figure 1. No signs of portal hypertension were observed; notably, the previously noted paraumbilical vein had disappeared, and the direction of blood flow in the portal vein was now normal (Figure 1). No ascites was present. Both liver and spleen stiffness decreased markedly (Table 2). The MRI based volumetry of liver showed a marked improvement of hepatomegaly (2631 ml, decrease of 26% vs. baseline) as seen in Figure 2 and a slight improvement of splenomegaly (554 ml, decrease of 9.6% vs. baseline) correlating with the ultrasound and clinical findings. Another year later, his clinical conditions are fully stable.

## Discussion

Amyloidosis is characterized by the abnormal extracellular accumulation of proteinaceous fibrillary material (amyloid) that is fairly resistant to normal proteolytic digestion. Systemic amyloidosis is subclassified by a particular precursor protein into two major forms, AL amyloidosis and secondary amyloidosis (AA amyloidosis) [3]. The primary form is characterized by amyloid deposits composed of the light chains fragment of immunoglobulin and it is associated with B-cell dyscrasia, particularly multiple myeloma, and monoclonal gammopathy. The secondary form is characterized by the deposition of an amyloid A protein (non-immunoglobulin) and it follows or co-exists with various disorders such as rheumatoid arthritis, tuberculosis, chronic long-standing infections or malignant tumors, particularly renal cell carcinoma [3].

The liver is a common site of amyloid deposition in primary AL amyloidosis. In an autopsy series [4], 70% of the patients with primary systemic amyloidosis had liver involvement, which is, however, most of the times asymptomatic. There are few reported cases of portal hypertension due to primary systemic amyloidosis [5-6]. Notably, liver involvement is associated with a reduced survival in patients with primary systemic amyloidosis (mean survival time: 10 to 14 months vs. 12 to 18 months in patients without liver involvement) [7-9] and the presence of portal hypertension further worsens patients’ outcome (mean survival time: eight to nine months) [10].

As in any severe disease, an early recognition of primary amyloidosis is crucial and liver involvement should be diagnosed for further risk stratification. Liver involvement can be suspected based on imaging findings, but its confirmation requires a liver biopsy. However, a higher risk of bleeding in patients with amyloidosis has been reported [11]. This has been ascribed to different factors: a) reduced synthesis and/or malabsorption of vitamin K-dependent clotting factors resulting in coagulopathy; b) rapid clearance of factor X due to binding to amyloid fibrils, resulting in a deficiency of factor X [12]; c) decreased contractility of vessels infiltrated by amyloid, which, once lacerated, may not clot normally [13-15].

Transjugular liver biopsy is a safe alternative in case of increased bleeding risk [16]. In our patient, this route was chosen before suspecting the diagnosis of amyloidosis due to the concomitant presence of portal hypertension.

In our patient, the finding of an increased alcohol consumption initially suggested a diagnosis of cirrhosis. This was questioned due to the presence of large hepatomegaly and abnormally high liver stiffness measurement. Interestingly, 2D-SWE (a modern ultrasound elastography technique which provides B-mode imaging in addition to elasticity measurements, and which can be performed in patients with ascites) [1] demonstrated extremely high values (mean 99.4 kPa), which are compatible with, but not typical of cirrhosis (usually not exceeding 70-75 kPa). Two previous cases have reported extremely high values of liver stiffness measured either with TE or with SWE [17-18], similar to the ones observed in our patient.

Our case is the first demonstrating that a VGPR to chemotherapy, likely decreasing liver amyloid content, was associated to a decrease in liver stiffness measured by TE and 2D-SWE, and was accompanied by a disappearance of signs of portal hypertension. Notably, liver stiffness improved but remained higher than normal (and in a range that could be still compatible with cirrhosis), but spleen stiffness measurement (SSM) completely normalized after treatment. SSM is increasingly recognized as a sensitive and specific non-invasive surrogate of portal pressure [2], with improved accuracy as compared to LSM.

## Conclusions

We have described the case of a patient presenting with ascites due to portal hypertension. We report how non-invasive diagnostic methods helped in the characterization of the cause of the syndrome. The presence of a very high liver stiffness and of patency of the paraumbilical vein suggested an intrahepatic cause of the syndrome. However, some characteristics such as a large hepatomegaly and an exceedingly high value of liver stiffness were not typical of cirrhosis (the most common cause of intrahepatic portal hypertension). Liver biopsy was determinant in diagnosing primary amyloidosis, and this underlines how non-invasive and invasive diagnostic methods provide complementary information in difficult cases. After the identification of hepatic amyloidosis, our patient was diagnosed with multiple myeloma and underwent chemotherapy. He showed a VGPR to treatment, and the clinical complications of portal hypertension disappeared. In parallel, non-invasive signs of the syndrome improved. This case, whose good outcome is not expected (primary amyloidosis presenting with portal hypertension usually has an ominous prognosis) shows that imaging and liver and spleen stiffness are useful techniques to be used in patients with portal hypertension on diagnosis and might be used as non-invasive biomarkers to follow-up patients with liver involvement due to primary systemic amyloidosis.
